# Asthenopia in Postgraduate Surgical Trainees: Prevalence and Correlation With Refractive and Orthoptic Parameters

**DOI:** 10.22599/bioj.481

**Published:** 2026-04-09

**Authors:** Aashi Bansal, Sakshi Meshram, Shreya Thatte, Shreya Mahanaik, Anupriya Kesharwani

**Affiliations:** 1 Department of Ophthalmology, SAMC & PGI, Indore, India

**Keywords:** Asthenopia, Resident surgeons, convergence, Refractive error

## Abstract

**Purpose:**

Association of asthenopia with multiple professions involving prolonged near work is well-established but asthenopia is under-reported in postgraduate surgical trainees. The purpose of this study was to determine the prevalence of asthenopia in surgical trainees and determine the associated risk factors.

**Methods:**

This study was an institute-based observational study, conducted between July 2024 and September 2024. Out of 107 surgical trainees, 102 responded to a structured asthenopia questionnaire. Detailed ophthalmic evaluation was performed in 43 trainees who consented and presented voluntarily. Visual acuity (Snellen), cover test (near and distance), positive (PFV) and negative fusional vergence (using a prism bar at near and distance), stereoacuity (Randot), near point of convergence, near point of accommodation and accommodative amplitude (using the Royal Air Force rule) were measured. Statistical analysis was performed using SPSS 22.

**Results:**

The prevalence of asthenopia was found to be 67.6%. The highest prevalence was found in Ophthalmology trainees. Out of 43 who presented for orthoptic assessment, 11 were advised to pursue refractive correction. PFV near was significantly lower in trainees with asthenopia (mean = 10.1 PD) compared to those without asthenopia (mean = 15.1), p = 0.03.

**Conclusion:**

Asthenopia among surgical trainees was high. The visual precision needed for surgical procedures, lack of sleep and the extensive academic curriculum may contribute to asthenopia in surgical trainees. Routine ophthalmic and orthoptic testing may help alleviate symptoms.

## Introduction

In the era of constant technological advancement and digitalisation, asthenopia or eye strain has drastically increased among all age groups, most notably in young adults (Hashemi *et al.*, 2019; Wang *et al.*, 2022; Ganne *et al.*, 2020; Azmoon *et al.*, 2013; Mohan *et al.*, 2021). This typically presents as headache, eye ache, blurring of vision, diplopia, a burning sensation or tiredness of eyes after sustained fixation at a close working distance. Suggested associated risk factors for asthenopia include ocular disorders (refractive error, dry eyes and binocular vision disorders), psychological factors like stress, screen use for long period of time, poor ambient light conditions while performing near work and lack of sleep (Wang *et al.*, 2022; Azmoon *et al.*, 2013; Schellini *et al.*, 2016; Han *et al.*, 2013). The resultant ocular and systemic distress can affect overall wellbeing, work capacity and productivity. A high prevalence of asthenopia has been documented in school and college students, and undergraduate medical students in particular (Hashemi *et al.*, 2019; Han *et al.*, 2013; Touma Sawaya *et al.*, 2020; Singh *et al.*, 2016), as well as in association with professions like banking officials, engineers, IT professionals and tailors (Touma Sawaya *et al.*, 2020; Comério *et al.*, 2017; Zafar *et al.*, 2022; Widia *et al.*, 2021). A high prevalence of asthenopia has been reported in radiologists, nurses and ophthalmologists (Alhasan & Aalam, 2022; Azmoon *et al.*, 2013; Lin *et al.*, 2023).

In surgical practice, visual demands are especially intense. Laparoscopic, endoscopic and microscopic procedures require fine hand–eye coordination, steady accommodation and accurate depth perception (Widia *et al.*, 2021). These are often performed under time pressure, during long shifts and with irregular rest, all while managing heavy academic commitments (Alhasan & Aalam, 2022). Under such conditions, surgical trainees may be particularly susceptible to visual fatigue. In early training, when procedural skills are still developing, unaddressed asthenopia may undermine concentration, manual precision and confidence. Despite this, little is known about how common asthenopia is among surgical trainees or which factors place them at higher risk. This study aimed to estimate the prevalence of asthenopia and explore associated risk factors, including refractive error and binocular function, among surgeons undergoing training in six surgical specialties: General Surgery, Obstetrics and Gynaecology (OBG), Ophthalmology, Otorhinolaryngology (ENT), Oral and Maxillofacial Surgery (OMFS) and Orthopaedics.

## Methods

**Figure 1:**
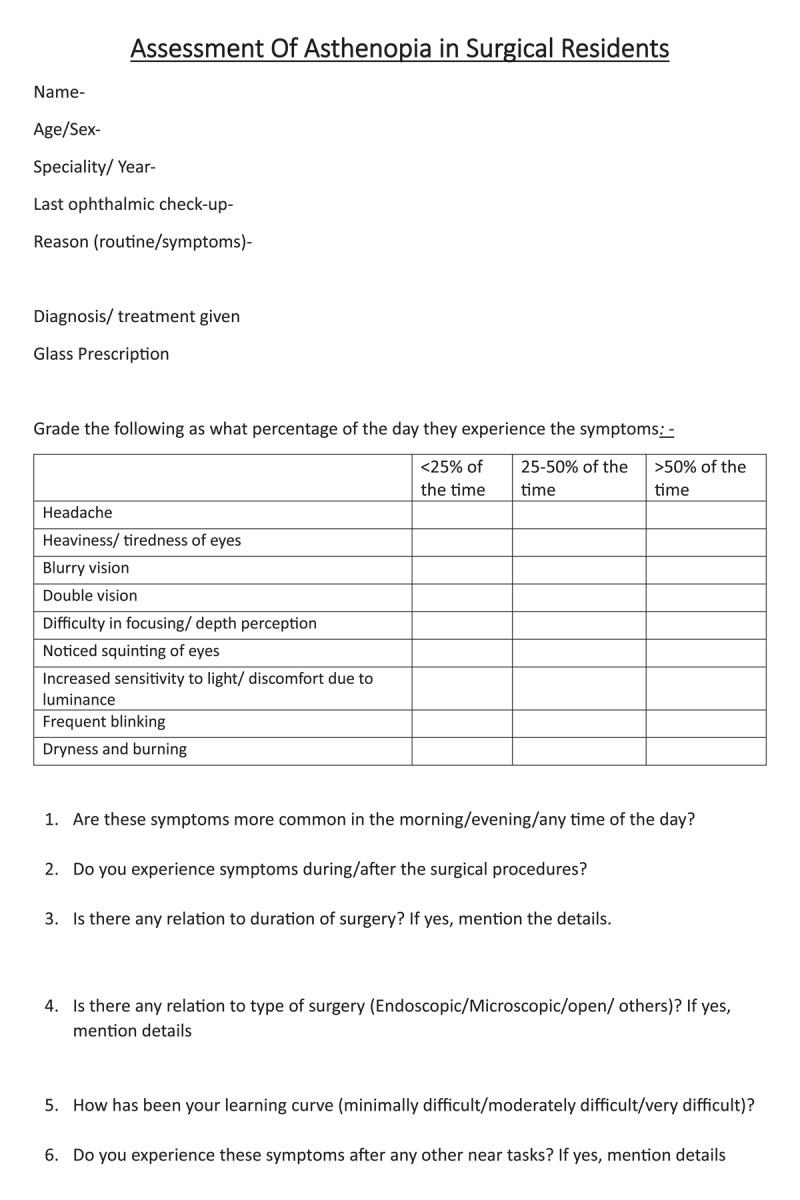
Asthenopia questionnaire.

This institute-based, cross-sectional study was conducted from July to August 2024 following approval from the Institutional Review Board (IEC NO: SAIMS/IEC/36/24). All postgraduate trainees enrolled in Ophthalmology, OBG, General Surgery, Orthopaedics, ENT and OMFS were invited to participate. Of 107 eligible trainees, 102 (95.3%) completed a structured questionnaire (Figure 1) capturing demographics; timing and details of the last ophthalmic examination including spectacle prescription; and the presence, frequency, timing and activity-specific triggers of visual symptoms. Asthenopia was operationally defined as the presence of ≥1 symptom for >50% of waking hours or ≥2 symptoms for >25% of waking hours. Symptoms queried included headache, ocular tiredness, blurred or double vision during near work, squinting, photophobia, frequent blinking and burning of eyes. Symptom frequency was graded to derive a score (Figure 1) (Sheedy *et al.*, 2003; Hayes *et al.*, 2007; Rosenfield, 2011; Blehm *et al.*, 2005).

All respondents were invited for comprehensive ophthalmic and orthoptic evaluation in the Ophthalmology clinic. Those that voluntarily attended the clinic provided informed consent for examination. Distance visual acuity (unaided and with habitual correction) was measured using a Snellen chart; near visual acuity was measured using a Jaeger chart. Ocular alignment was evaluated using the cover–uncover and alternate cover tests for distance (6 m) and near (33 cm); the magnitude of phoria was measured using a prism cover test (with a prism bar). Positive (PFV) and negative fusional vergences (NFV) to break point and recovery point were recorded at distance and near (with a prism bar). Stereoacuity was measured using the Randot near stereo test. Near point of convergence (NPC), near point of accommodation (NPA) and accommodative amplitude (AA) were measured with the Royal Air Force rule using a 6/9 equivalent target. Refraction was performed in participants with unaided or habitual distance visual acuity worse than 6/6 or in those reporting asthenopic symptoms. Detailed examination of anterior (using slit lamp) and posterior segment (using indirect ophthalmoscope) of the eye was performed.

Data were entered into Microsoft Excel (2010) and analysed using SPSS (version 22). Quantitative variables are presented as mean (standard deviation, SD), and categorical variables as frequency (percentage). Group comparisons for age, sex, asthenopia score and stereoacuity were performed using independent-samples t-tests. Bonferroni correction was used for multiple comparisons. Pearson correlation coefficients assessed associations between the asthenopia score, NPC, NPA, AA, PFV and NFV. Statistical significance was set at p < 0.05.

All trainees in the six targeted surgical disciplines were approached, effectively constituting a census of the eligible population during the study period. No exclusions were applied other than non-response to the questionnaire or lack of consent for clinic evaluation. The questionnaire was pilot-tested for clarity among a small group, and minor wording refinements were made before deployment. Although formal reliability testing (e.g., internal consistency) was not undertaken, items were adapted from previously used instruments in studies of asthenopia and digital eye strain (Sheedy *et al.*, 2003; Hayes *et al.*, 2007; Rosenfield, 2011; Blehm *et al.*, 2005). To minimise measurement bias, the same examiners conducted refraction and orthoptic testing using a standardised protocol and calibrated equipment. All assessments were performed in a fixed sequence without randomisation or counterbalancing: distance and near visual acuity; stereoacuity; cover–uncover and alternate cover tests with prism bar measurement; positive and negative fusional vergences at distance and near; near point of convergence, near point of accommodation and accommodative amplitude; refraction when indicated and, finally, anterior and posterior segment evaluation. For vergence and accommodation testing, participants were instructed using a scripted set of directions to ensure consistent understanding of the task. Where refraction was repeated, the final correction was determined by manifest refraction aligned with best subjective acuity. Questionnaires with incomplete symptom frequency data were excluded from asthenopia scoring but retained for descriptive statistics when appropriate; no imputation was performed. Continuous variables met approximate normality assumptions on visual inspection of histograms; hence, t-tests were used for group comparisons. Correlations were interpreted with attention to clinical, not merely statistical, significance.

## Results

**Figure 2:**
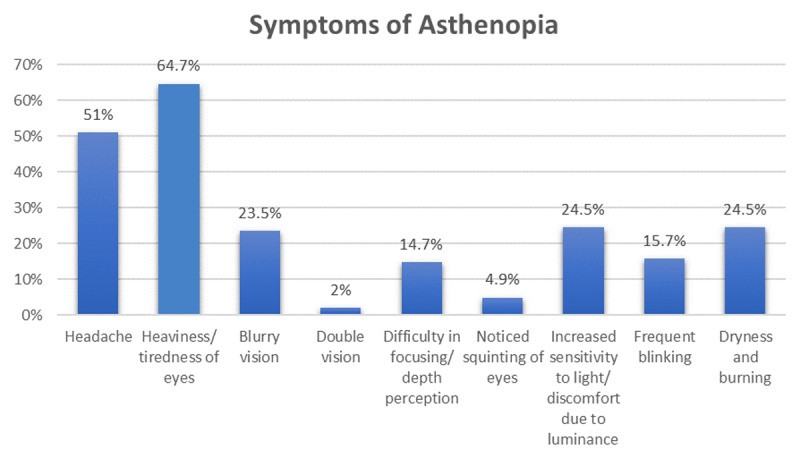
Bar diagram showing frequency of various symptoms of asthenopia in postgraduate surgical trainees.

Of the 107 surgical trainees approached, 102 (95.3%) completed the questionnaire and were included in the analysis. The mean age of respondents was 27.3 years (range 24–34); 47 (46.1%) were female and 55 (53.9%) were male. Overall, 69 surgical trainees (67.6%) met the case definition for asthenopia, with a mean asthenopia score of 2.41 (SD 2.10). Twenty-two affected trainees (27.5%) reported evening exacerbation of symptoms, and 19 (22.5%) noted symptom aggravation after surgical procedures. The most frequently reported symptom was ocular tiredness (64.7%), followed by headache (Figure 2).

Asthenopia was more prevalent among female trainees, who also had a higher mean asthenopia score than males (females 3.32 vs males 1.60; p < 0.001; Table 1). By specialty, the highest prevalence was observed in Ophthalmology trainees (91.6%), who also had the highest mean score (4.3) (Table 1).

**Table 1:** Gender and specialty distribution and mean asthenopia scores among postgraduate surgical trainees.


PARAMETER	SUBGROUP	TOTAL (n)	WITH ASTHENOPIA (n, %)	WITHOUT ASTHENOPIA (n, %)	MEAN ASTHENOPIA SCORE

**Gender**	Females	47	40 (85.1%)	7 (14.9%)	3.32

	Males	55	29 (52.7%)	26 (47.3%)	1.60

**Specialty**	Ophthalmology	12	11 (91.6%)	1 (8.4%)	4.30

	ENT & OMFS	18	13 (72.2%)	5 (27.8%)	2.53

	OBG	18	13 (72.2%)	5 (27.8%)	2.83

	Orthopaedics	26	15 (57.6%)	11 (42.4%)	1.69

	Surgery	28	17 (50.0%)	11 (50.0%)	1.96


Forty-five trainees (44.1%) reported a prior diagnosis of refractive error (spherical equivalent +0.50 to –7.30 D). Simple myopia predominated (n = 36), followed by myopic astigmatism (n = 7), hyperopia (n = 1) and astigmatism (n = 1). Asthenopia was present in 80.0% of ametropic trainees (mean score 3.1) compared with 57.8% of emmetropes (mean score 1.8).

Of the 102 survey respondents, 43 (42.2%) attended the outpatient clinic for detailed assessment; 30 of 43 (69.7%) met the asthenopia criterion. Clinic attendance was proportionally higher among trainees from Ophthalmology (66.7%), ENT and OMFS (72.2%) than from General Surgery (19.2%) or Orthopaedics (17.9%), which is consistent with the greater frequency of visually demanding microscopic or endoscopic procedures in the former specialties.

Among the 43 examined trainees, 34 had distance visual acuity of 6/6, either unaided or with current spectacles, while the remaining nine had refractive errors. Seven trainees were prescribed a change in spectacle power and two were newly identified as having astigmatism. On cover testing, 40 trainees were either orthophoric or had small exophoria (<4 PD). Three had larger exophorias (10–12 PD); one required an updated refractive correction and two were diagnosed with convergence insufficiency (based on receded near point of convergence and reduced near positive fusional vergence). Detailed orthoptic testing was completed in the 34 evaluated participants, of whom 23 (67.6%) had asthenopia. Mean near PFV at break was significantly lower in participants with asthenopia than in those without asthenopia (12.39 PD vs 18.18 PD; p = 0.030). Mean near PFV at recovery was likewise reduced in the asthenopia group (10.13 PD vs 15.09 PD; p = 0.033).

All other orthoptic parameters were comparable between those with and without asthenopia. No statistically significant differences were observed in NPC, NPA, AA, distance PFV or NFV (Table 2).

**Table 2:** The average values of various orthoptic parameters amongst resident surgeons with asthenopia and without asthenopia. Abbreviations: NPC = near point of convergence; NPA = near point of accommodation; AA = accommodative amplitude; PFV = positive fusional vergence; NFV = negative fusional vergence; D = distance, 6 m; N = near, 33 cm; BP = break point; RP = recovery point; PD = prism dioptres.


ORTHOPTIC PARAMETERS (MEAN)	WITH ASTHENOPIA	WITHOUT ASTHENOPIA	P VALUE OF DIFFERENCE

NPC (cm)	7.13	7.64	0.597

NPA (cm)	7.70	6.91	0.413

AA	13.61	14.82	0.379

PFV-D(BP) [PD]	10.04	12.27	0.361

PFV-D (RP) [PD]	7.96	10.00	0.347

NFV-D(BP) [PD]	7.74	9.64	0.157

NFV-D (RP) [PD]	5.74	7.64	0.157

PFV-N(BP) [PD]	12.39	18.18	0.030

PFV-N (RP) [PD]	10.13	15.09	0.033

NFV-N(BP) [PD]	11.61	11.45	0.901

NFV-N (RP) [PD]	9.48	9.45	0.983


^1^*Bonferroni correction was applied across all orthoptic parameters compared between the two groups*.

There was no statistically significant difference between male and female participants in orthoptic parameters (all p > 0.05). Stereoacuity ranged from 20 to 100 arcsec, with a mean of 56.3 arcsec; there was no significant sex difference (females 58.3 arcsec vs males 54.3 arcsec) and no significant association between stereoacuity and asthenopia (p = 0.11).

## Discussion

The prevalence of asthenopia in this cohort of postgraduate surgical trainees (67.6%) aligns with previously reported rates across student and professional populations, which vary widely from 40% to 90% (Azmoon *et al.*, 2013; Mohan *et al.*, 2021; Schellini *et al.*, 2016; Kandel *et al.*, 2017; Han *et al.*, 2013; Touma Sawaya *et al.*, 2020; Singh *et al.*, 2016; Comério *et al.*, 2017; Zafar *et al.*, 2022; Widia *et al.*, 2021; Alhasan & Aalam, 2022; Lin *et al.*, 2023). Studies of university students have reported similarly high prevalence, as seen in Lebanon (67.8%) (Touma Sawaya *et al.*, 2020), Egypt (86%) (Iqbal *et al.*, 2018) and Kathmandu (90.8%) (Shrestha *et al.*, 2023). Comparable observations have been made in clinical groups, including ophthalmologists, where rates range from 40% to over 90% depending on workload and setting (Dzhodzhua *et al.*, 2017; Lin *et al.*, 2023).

Our findings indicate a greater prevalence and severity of asthenopia among female participants, in agreement with several earlier reports (Touma Sawaya *et al.*, 2020; Hashemi *et al.*, 2019). Nevertheless, the literature remains inconsistent, as other studies have not identified a significant sex-related difference in asthenopia (Han *et al.*, 2013; Wang *et al.*, 2022).

Ametropia emerged as a significant associated factor, particularly myopia. This aligns with reports showing increased asthenopia symptoms in myopes engaged in prolonged near work and the potential benefit of structured breaks or optimised viewing distances (Wang *et al.*, 2022). By contrast, Kiati *et al.* (2022) did not find a significant association between refractive error and asthenopia among clinical microscopists, although they documented strong links between visual fatigue and orthoptic parameters such as AA, NPC and near PFV.

Specialty-specific patterns were notable. Trainees in Ophthalmology, ENT and OMFS—fields requiring frequent microscopic or endoscopic work—reported more severe symptoms. These tasks impose sustained demands on convergence and accommodation. Our finding of reduced near PFV among symptomatic trainees supports a possible mechanism of vergence stress during high-demand near viewing. Similar associations have been highlighted in clinical microscopists (Kaiti *et al.*, 2022). Conversely, no significant association was found between stereoacuity and symptoms, which aligns with mixed evidence regarding stereoacuity’s contribution to visual discomfort (Kim *et al.*, 2013).

Workload-related effects have been described elsewhere; for instance, ophthalmologists exhibit greater visual fatigue after outpatient or operative sessions, with improvement during periods of leave (Dzhodzhua *et al.*, 2017). Night-shift related reductions in accommodative response have also been documented (Masuda *et al.*, 2005). Such findings contextualise the high prevalence observed in trainees, whose schedules often involve long hours, irregular sleep, and repetitive near-demand tasks.

Addressing asthenopia is important from a training and safety perspective. Unmanaged symptoms may impair concentration and slow psychomotor skill acquisition during procedures. Practical measures to minimise symptoms include regular refraction updates, optimising microscope and monitor ergonomics, ensuring adequate illumination and introducing scheduled brief breaks during periods of sustained near work. Trainees with reduced near PFV or evidence of convergence insufficiency may benefit from focused vergence or accommodative exercises.

A strength of this study is its focus on a rarely examined population—postgraduate surgical trainees—combined with a structured binocular vision assessment for a subset of participants. Nonetheless, several limitations warrant consideration. The cross-sectional design limits causal interpretation. The clinical evaluation subset (n = 43) may have introduced selection bias if symptomatic trainees were more likely to attend. Asthenopia was assessed using symptom-frequency thresholds rather than a validated composite instrument, and potentially relevant variables such as sleep duration, screen time and ergonomic factors were not quantified. The single-centre setting may also limit generalisability to other training environments.

Future work should incorporate multi-centre, longitudinal designs to evaluate symptom progression across rotations and quantify near work exposure, sleep patterns and operative workload. Randomised evaluations of ergonomic modifications or orthoptic interventions could help identify effective strategies to reduce symptoms. Understanding the relationship between visual fatigue and surgical performance metrics may further inform evidence-based policies for trainee wellbeing. Given the demands of residency training, brief screening tools may be more feasible than lengthy questionnaires. Embedding a concise symptom-frequency checklist into annual occupational health assessments could support early identification of trainees who may benefit from refraction updates or orthoptic evaluation.

## Conclusion

Asthenopia was common among postgraduate residents in surgical specialties and appears to be higher in visually intensive disciplines, such as Ophthalmology. Female sex, ametropia and reduced near PFV were associated with worse asthenopia symptoms. Routine ophthalmic screening with annual refraction and orthoptic evaluation when indicated may mitigate symptoms and support safer, more effective surgical training.

## Data Accessibility Statement

The data supporting the findings of this study are available from the corresponding author upon request.
